# The subpolar gyre regulates silicate concentrations in the North Atlantic

**DOI:** 10.1038/s41598-017-14837-4

**Published:** 2017-11-06

**Authors:** H. Hátún, K. Azetsu-Scott, R. Somavilla, F. Rey, C. Johnson, M. Mathis, U. Mikolajewicz, P. Coupel, J.-É. Tremblay, S. Hartman, S. V. Pacariz, I. Salter, J. Ólafsson

**Affiliations:** 1 Faroe Marine Research Institute, Box 3051, FO-110 Torshavn, Faroe Islands; 2 Institute of Earth Sciences, University of Iceland, Reykjavik, Iceland; 3 Bedford Institute of Oceanography, Department of Fisheries and Oceans, Dartmouth, Nova Scotia Canada; 4 Instituto Español de Oceanografía, Madrid, Spain; 5 Institute of Marine Research, c/o Department of BioSciences, University of Oslo, Oslo, Norway; 6 SAMS, Scottish Marine Institute, Oban, Argyll Scotland; 7 Max Planck Institute for Meteorology, Hamburg, Germany; 8 Québec-Océan and Takuvik, Département de Biologie, Laval University, Quebec, Canada; 9 National Oceanography Centre, Southampton, UK; 10 Department of Marine Sciences, University of Gothenburg, Gothenburg, Sweden; 11 Laboratoire d’Océanographie Microbienne (LOMIC), Observatoire Océanologique, Sorbonne Universités, UPMC Univ Paris 06, CNRS, 66650 Banyuls-sur-Mer, France

## Abstract

The North Atlantic is characterized by diatom-dominated spring blooms that results in significant transfer of carbon to higher trophic levels and the deep ocean. These blooms are terminated by limiting silicate concentrations in summer. Numerous regional studies have demonstrated phytoplankton community shifts to lightly-silicified diatoms and non-silicifying plankton at the onset of silicate limitation. However, to understand basin-scale patterns in ecosystem and climate dynamics, nutrient inventories must be examined over sufficient temporal and spatial scales. Here we show, from a new comprehensive compilation of data from the subpolar Atlantic Ocean, clear evidence of a marked pre-bloom silicate decline of 1.5–2 µM throughout the winter mixed layer during the last 25 years. This silicate decrease is primarily attributed to natural multi-decadal variability through decreased winter convection depths since the mid-1990s, a weakening and retraction of the subpolar gyre and an associated increased influence of nutrient-poor water of subtropical origin. Reduced Arctic silicate import and the projected hemispheric-scale climate change-induced weakening of vertical mixing may have acted to amplify the recent decline. These marked fluctuations in pre-bloom silicate inventories will likely have important consequences for the spatial and temporal extent of diatom blooms, thus impacting ecosystem productivity and ocean-atmosphere climate dynamics.

## Introduction

The subpolar North Atlantic is characterized by deep winter convection^1^, and a strong diatom dominated spring bloom^2^. Diatoms are fast-growing algae that in addition to phosphate and nitrate require silicic acid, hereafter referred to as silicate, to sustain their growth^3^. In the subpolar North Atlantic, silicate is the main limiting nutrient for diatom growth^4^ although occasionally outweighed by seasonal iron limitation^5^. Diatoms are an important food source for secondary producers, and in particular for calanoid copepods such as *Calanus finmarchicus* ^6^, which itself is a key prey item linking microbial components to higher trophic levels in subpolar ecosystems^7^.

**Figure 1:**
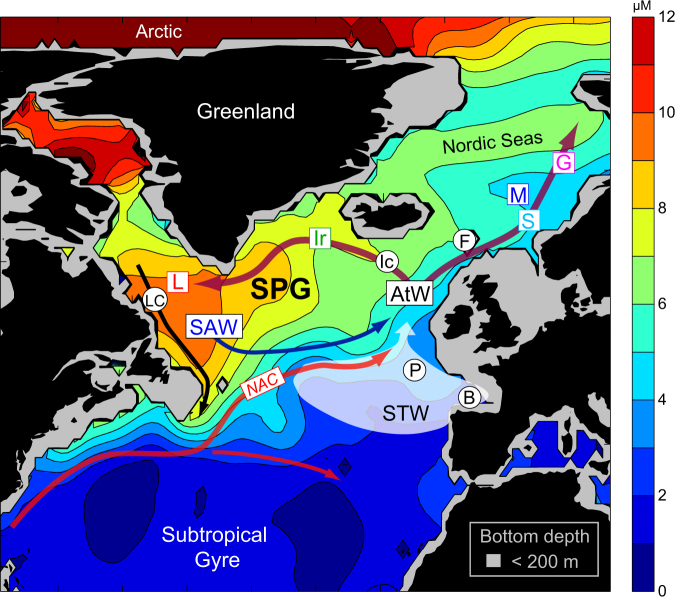
Schematic of the emphasized features in the subpolar Atlantic, with the climatological average upper ocean (0–200 m) silicate concentrations during April obtained from the World Ocean Atlas^20^ contoured with colors. Oceanographic abbreviations: Subpolar gyre (SPG), SubArctic Water (SAW), SubTropical Water (STW), Atlantic Water (AtW) and the North Atlantic Current (NAC). The silicate observations shown in Fig. 2a are sampled in the central Labrador Sea (L), the northern Irminger Sea (Ir), and in the Nordic Seas – the Svinøy Section (S), Ocean Weather Ship M (M) and the Gimsøy Section (G) (colored letters). Supporting time series (Supplementary Information 1, black letters) are obtained from the Porcupine Abyssal Plain (P) and the southern Bay of Biscay (B) (Fig. 3), the Labrador Current (LC) (Fig. 5), the Iceland Basin (Ic) and the Faroe Shelf (F). The figure was produced using the software Matlab R2013b, (https://www.mathworks.com).

Along its eastern boundary, the subpolar North Atlantic receives relatively warm and saline water from the subtropical gyre characterized by silicate concentrations of less than 3 µM, averaged over the upper 200 meters (Fig. 1). The productivity of the subpolar ocean is therefore crucially dependent on both deep winter convection and silicate import from the Arctic. Arctic water, which is rich in silicate^8^, enters the Atlantic through the Canadian Arctic Archipelago and in smaller volumes through the Fram Strait, and it is carried equatorward by the Labrador Current^9^. Silicate also accumulates in the deep Overflow Waters that pass through the Labrador Sea and, together with Labrador Sea Water, continue southwards as the Deep Western Boundary Current^1^. The counterclockwise rotating subpolar gyre (SPG) prevents these silicate-rich waters from draining southwards out of the subpolar region. A fraction of the Labrador Sea Water and a part of Labrador Current water, here collectively referred to as Sub Arctic Water (SAW), are flowing eastwards along the northern edge of the North Atlantic Current^10^ and admixed with the nutrient poor subtropical waters west of the British Isles^11^ (Fig. 1). Western waters from the Gulf Stream and eastern waters from the Bay of Biscay region are hereafter jointly discussed as Sub Tropical Water (STW). The confluence of SAW and STW produces silicate enriched Atlantic Water (AtW), which flows poleward through the Nordic Seas and northwestward towards Greenland (Fig. 1), thus retaining a portion of the silicate within the subpolar domain.

The size and circulation strength of the SPG is highly variable^11^ and depends on wintertime atmospheric forcing, primarily air-sea heat exchange and wind stress curl^12^. This variability is represented by the unitless *gyre index* calculated from the sea surface height field^11,12^ (see Methods) which, in turn, represents surface currents, the buoyancy content and the position of major fronts in the North Atlantic^13^. Weak atmospheric forcing is associated with shallow winter mixing (depths), a westward retraction of the SPG and a weak and southward shifted North Atlantic Current with decreased eastward transport of SAW and a low gyre index. This physical regime opens a ‘window’ between the SPG and the European Continental slope increasing the admixture of nutrient poor STW to the poleward AtW flow. The gyre index therefore explains the relative SAW and STW mixture to the AtW^11^.

In the present study a new 25-year (1990–2015) compilation of pre-bloom silicate observations is analyzed, gathered from several independent hydrographic sections crossing the Atlantic Inflow branches in the Nordic Seas, the northern Irminger Sea and the central Labrador Sea (Fig. 1). Our records represent the vertically homogenous winter mixed layer, and thus a water column of typically more than a kilometre in the west, and several hundred meters along the eastern side. Considering that the diatom-dominated spring bloom in the North Atlantic becomes silicate limited every year, these time-series of pre-bloom silicate concentrations represent long-term trends in the capacity for diatom related primary production throughout a huge volume in the subpolar North Atlantic Ocean. The data compilation includes series from three long-term observation sites along the Norwegian slope that have been updated from previous work^14^, and incorporates previously unpublished series from the Labrador and Irminger Sea (Methods and Supplementary Information 1). These *primary* datasets, which have been consistently sampled during the winter/early spring months in order to reflect pre-bloom concentrations in the winter mixed layer (Methods), are illustrated with coloured squares in Fig. 1. We also present *supporting* silicate time series from the Labrador Shelf, the Iceland Basin, the Faroe Shelf, the Porcupine Abyssal Plain and the Bay of Biscay (Methods and Supplementary Information 1) - shown with black circles in Fig. 1.

The observations are compared with output from a global model system consisting of the ocean general circulation model MPIOM (Max-Planck-Institute Ocean Model)^15^ and the marine biogeochemistry model HAMOCC (Hamburg Ocean Carbon Cycle Model)^16^, driven with ERA40^16^ and ERA-Interim^17^ reanalysis data. More details are provided in Methods and Supplementary Information 1. Previous studies have demonstrated that simulated fields from MPIOM compare favourably with observations in the Labrador and Irminger Seas^13^.

**Figure 2:**
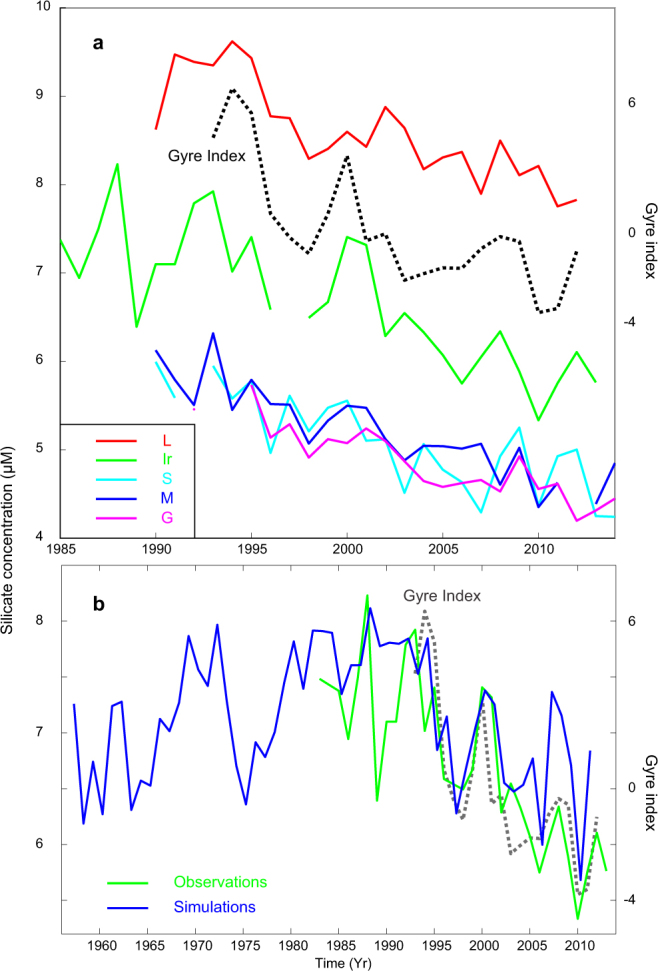
Temporal evolution of key parameters. (**a**) Colored lines show pre-bloom upper ocean silicate concentrations across the subpolar Atlantic; L, Ir, S, M and G in Fig. 1. The samples are made in the pre-bloom homogeneous winter mixed layer, and thus represent several hundred meters in the Nordic Seas and typically more than a kilometre in the Labrador Sea (see Methods). The dashed black line shows the unitless gyre index, associated with the leading North Atlantic sea-surface height mode, as obtained from altimetry observations^41^ (Methods). (**b**) Similar to (**a**), but showing the silicate concentrations in the northern Irminger Sea (Ir in Fig. 1) for a longer time period. The observations are in green, simulations (0–200 m, March) in blue and the gyre index in dashed gray.

We observe a consistent decline of pre-bloom silicate concentrations throughout the subpolar North Atlantic of 1.5–2 µM over the 25-year observation period (Fig. 2a). The rate of decline is similar in the central Labrador Sea, the northern Irminger Sea and in the Nordic Seas, all occupying the range 0.55–0.66 µM per decade (Table 1 and Fig. 2a). Note that the supporting datasets from the Iceland Basin and Faroe Shelf exhibit comparable rates of decline over the same period (Table 1). These latter datasets exhibit more variability, likely due to variable sampling timing (Dec-Feb for the Faroe Shelf and differing summer months for the Iceland Basin, see Table S2) and locally complex coastal dynamics, and have thus merely been used to support the primary analysis. The consistency of the silicate decline over the large spatial domain from which the observations originate is remarkable and suggests that basin-scale physical mechanisms are responsible.

**Table 1:** Silicate trend analysis over the period 1990–2015. The abbreviated regions are shown in Fig. 1, and the statistics are described in Methods.

Region	µM/decade	*r* ^2^	*P*-value
L	−0.66	0.68	<1e-6
Ir	−0.66	0.63	<1e-6
S	−0.60	0.67	<1e-5
M	−0.61	0.78	<1e-7
G	−0.55	0.79	<1e-7
Ic	−0.78	0.37	0.021
F	−0.70	0.38	<0.01

In the following section we explore three non-exclusive physical mechanisms that could explain the observed silicate decline in the subpolar North Atlantic: (*i*) decreased concentrations in the already silicate-poor STW, (*ii*) shallower winter convection and a weakening SPG and (*iii*) decreased silicate influx from the Arctic.

**Figure 3:**
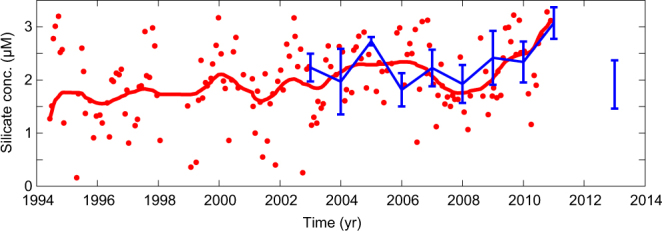
Silicate concentrations in the STW. Red dots show silicate concentration time-series from 150 m in the southern Bay of Biscay (B in Fig. 1), and the red line shows the 12 months low-pass filtered value. The blue line is the near-surface winter silicate concentrations from the region around the Porcupine Abyssal Plain (PAP) mooring (P in Fig. 1, March averages with standard errors).

To investigate Mechanism (*i*), we analysed previously unpublished observations from the eastern region occupied by STW (southern Bay of Biscay and the Porcupine Abyssal Plain, Fig. 1) and found no evidence of a persistent silicate decline (Fig. 3). In the Sargasso Sea, a weak near-surface (0–150 m) silicate decline was observed during the late 1990s, but this was followed by a subsequent increase^18^. Taken together, these data indicate that the silicate content in the STW has remained relatively constant over the observation period, ruling out this mechanism as an explanation for the observed decline in the subpolar region.

**Figure 4:**
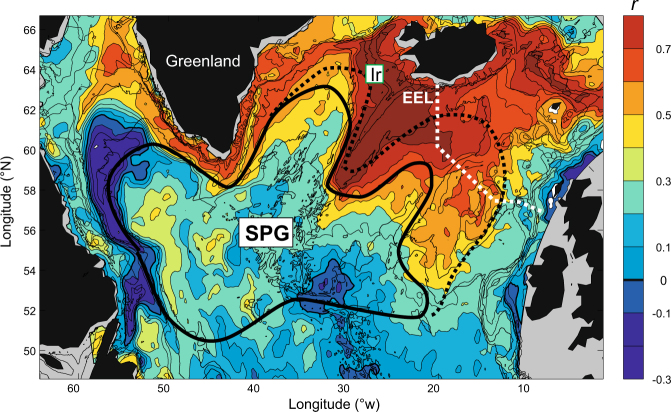
Analysis of the simulated (HAMOCC) near-surface (0–150 m) silicate during March. The map shows the correlation between the time series in the northern Irminger Sea (Ir, green box) and the times series at each individual model grid cell (1958–2011). The white dotted line shows the Extended Ellett Line (EEL) hydrographic section, and the black lines outline the approximate boundary of the subpolar gyre (SPG) during a weak (full) and strong (dashed) state. The figure was produced using the software Matlab R2013b, (https://www.mathworks.com).

To address the role of the SPG (Mechanism *ii*), we first note that a similar silicate decline is not observed in the Rockall Trough^19^, but the negative trend becomes increasingly identifiable when moving northwestward into the SAW within the SPG, approaching 1 µM per decade close to Iceland (Extended Ellett Line, Figs 4b and S1). Silicate concentrations in the Irminger Sea and Labrador Sea (Fig. 1) are highly correlated with the gyre index (Fig. 2a, Table 2) and thus mirror the major temperature and salinity increase since the early 1990s^11^, indeed suggesting a subpolar origin for the observed silicate decline.

**Table 2:** Silicate correlated against the gyre index (1993–2013). To investigate if the gyre index explains inter-annual variability, the series have also been de-trended by subtracting a linear trend line, prior to the correlation analysis. The abbreviated regions are shown in Fig. 1, and the statistics are described in Methods.

Region	Gyre Index (GI)	GI (de-trended)
	*r* ^2^, *P*	*r* ^2^, *P*
L	0.89, <1e-6	0.67, 0.002
Ir	0.82, <1e-4	0.51, 0.021
S	0.78, <1e-4	0.51, 0.021
M	0.78, <1e-4	—
G	0.78, <1e-3	0.49, 0.048

The MPIOM/HAMOCC model system is now utilized to examine the potential link between the SPG and the silicate variability in the subpolar Atlantic at a resolution that cannot be achieved from the observations alone. Climatological upper-ocean April silicate concentrations produced by the model system are in reasonable agreement with the World Ocean Atlas^20^ (Fig. S2). The model also captures the silicate decline since the early 1990s, although primarily along the westward retracting subarctic fronts, both along the periphery of the SPG and in the Nordic Seas (Fig. S3, Supplementary Information 2). This is likely because SAW is replaced by silicate poorer AtW, which renders pronounced changes along the frontal zones.

It has previously been shown that the shifting fronts associated with the SPG dynamics have an especially strong imprint on the hydrography and the ecosystem in the biologically productive Northern Irminger Sea^11,13,21^. HAMOCC adequately reflects the observed pre-bloom silicate variability in this key region, albeit with some discrepancy in the late 1980s (Fig. 2b, *r* = 0.80, *P* < 1e-6, see Methods for statistics). The simulated trend at site ‘Ir’ is comparable to the observations, and this trend is the focus of the present study.

The model furthermore shows realistic temporal variability in the central SPG (Fig. S4), although the location of the deep convection site is located somewhat southeast from the real convection region (Fig. S3), which is transected by WOCE line ARW7^22^.

With the limitations associated with specific model processes in mind, the upstream origin and the downstream imprint of the observed and simulated signal at site ‘Ir’ is further examined. We correlate the model-simulated pre-bloom near-surface silicate in the grid point nearest ‘Ir’ (averaged vertically over 0 to 150 m and temporally over March) with the similarly processed time series in all other model grid cells (Fig. 4). The silicate variability in the Irminger Sea does not correlate with areas south of the SPG, providing further evidence that the silicate trend does not originate in the subtropical Atlantic. However, strong correlations exist with the AtW mixing region of SAW and STW, as well as along the AtW flow pathways both towards the Arctic and cyclonically around the SPG and into the Labrador Sea. These analyses suggest that the mechanistic processes causing reductions in silicate concentration primarily operate in the AtW mixing region, which includes the Rockall-Hatton Plateau, the Iceland Basin and the Reykjanes Ridge (Fig. 4).

In the AtW mixing areas, the SAW is found at 500–800 m depths^23^, thus convective vertical mixing is required to entrain it into the upper waters. Strong positive correlations between the simulated upper layer silicate and winter mixed layer depths (both parameters averaged over December-February, Fig. S5) highlight that, the silicate variability in the subpolar North Atlantic is partly driven by convection and thus by air-sea heat exchanges. Atmospheric forcing primarily acts locally by cooling near-surface waters, which become dense and subsequently sink. In addition, there is a preconditioning effect from deep convection in the Labrador Sea, which expands the SPG into the Irminger Sea and Iceland Basin and, in turn, alters the water column density structure modulating convective processes. The post-1995 decline of the SPG size and circulation strength^11^ is intrinsically coupled with the decreasing winter convection depths (from nearly 2.5 km in 1994 to 600–800 m around 2010 in the Labrador Sea)^12^, and thus provides a mechanism whereby reduced vertical nutrient fluxes lead to upper ocean silicate decline.

The limited grid resolution of the model likely leads to an underestimated eddy driven influx of AtW from the boundaries of the Labrador Sea into the center of the Labrador Basin^24^ (Fig. 4). However, the observations demonstrate that the Labrador Sea silicate record is strongly correlated with that from the northern Irminger Sea (Fig. 2a, *r* = 0.86, *P* < 1e-6). It thus seems reasonable to conclude that the silicate variability that originates south of Iceland is advected into the Labrador Sea similarly to hydrographic anomalies in the region^11^. Further convection in this region enriches the AtW with silicate resulting in concentrations that are approximately 2 µM above the Irminger Sea values (Fig. 2a).

The Labrador Sea record can be considered to represent silicate variability in the SAW, the main body of the SPG itself. It is therefore likely that the declining silicate concentrations in the eastward flowing SAW source water will subsequently lower the silicate in the poleward flowing AtW – produced from the mixture of SAW and STW^11^. Additionally, since a weakening and contracting gyre acts to increase the proportion of silicate-poor STW to the mixed AtW^11,25^ this mechanism acts synergistically with weaker convective processes to contribute to the observed AtW silicate decline.

Based on the presented observations and the analysis of model simulation, we suggest that SPG dynamics and winter convection (Mechanism *ii*) offer a mechanistic framework to explain declining silicate concentrations through (a) variable vertical fluxes of silicate rich deep-water, (b) SPG regulation of SAW source water concentrations and (c) the relative SAW/STW contribution to the mixed AtW west of the British Isles (Fig. 1).

**Figure 5:**
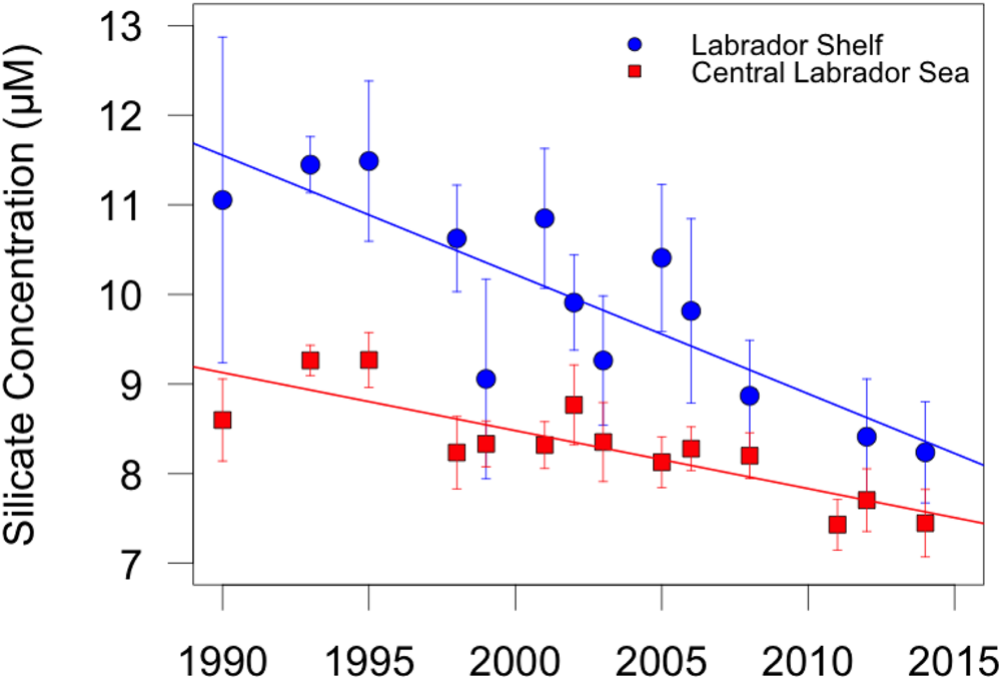
Temporal evolution of the upper-ocean silicate concentrations across the Labrador Shelf which has entered through the Canadian Arctic Archipelago (LC in Fig. 1, blue) and the central Labrador Sea (L in Fig. 1, red). The plot includes data from the 50–150 m layer for the Labrador Shelf and 150–300 m for the central Labrador Sea (averages with standard errors).

Finally, we explore whether any variability in inputs from the Arctic could contribute towards the observed silicate decline in the subpolar North Atlantic (Mechanism *iii*). Silicate concentrations on the Labrador Shelf (Methods and Supplementary Information 1), which include water that has entered the region through the Canadian Arctic Archipelago^9^, are also declining and at twice the rate observed in the central Labrador Sea (Fig. 5). Indication of declines in silicate concentrations are also observed further North at the main entry points of Silicate-Rich Arctic Water (SRAW)^11^ into Baffin Bay and the Labrador Sea (Fig. S6). Concurrently with the decline of the SPG during the mid-1990s, the Arctic circulation shifted to a persistently anticyclonic regime reflected in a positive phase of the Arctic Ocean Oscillation^26^. This results in increased retention of SRAW in the Beaufort Gyre and the Canada Basin^27^ of 8,000–12,000 km^3^ since the mid-1990s^28,29^. The evolution of SPG dynamics and the Arctic Ocean Oscillation could thus further contribute to the observed silicate decline.

In order to estimate the magnitude of Arctic influence, an approximate mass balance is presented in Supplementary Information 2 (Fig. S7). Since the silicate concentrations in the SRAW and the mean concentrations of the Arctic are 22 µM^8^ and 11 µM, respectively, it is estimated that about 8.8–14.4 ·10^16^ µmol must have accumulated in the Beaufort Gyre and Canada Basin since the mid-1990s. Between 85% and 100% of the silicate flux from the Arctic reaches the North Atlantic, mainly in the density range σ_θ_ = 27.0–27.59, and this encompasses a volume of about 4 ·10^14^ m^3^. The silicate decrease in the subpolar North Atlantic Ocean, caused by the retention of silicate in the Arctic Ocean since the mid-1990s is therefore 0.2–0.3 µM, which is <15% of the observed silicate decline of 1.5–2 µM (Fig. 2). We conclude that variability in silicate input from the Arctic leaves a weak imprint on the dominant SPG mechanism described above, although further silicate transport observations would be required to accurately partition the quantitative contribution.

Silicate decline over such a large area in the North Atlantic is likely to have a significant impact on ecosystem structure and climate dynamics. Theoretically, pre-bloom silicate concentrations exert a first order control on the maximum potential of diatom production during the spring bloom. There is some evidence from the Continuous Plankton Recorder surveys^30^ conducted during 1991–2009 that the relative contribution of diatoms to total phytoplankton abundance is decreasing in the North Atlantic^31^ in line with the observed silicate decline presented here. This would clearly affect standing stocks of calenoid copepods linking primary productivity to higher trophic levels, including commercially important fish and seabird populations. Shifts in phytoplankton community composition to lightly silicified and non-silicified phytoplankton may occur earlier in the growth season, potentially influencing copepod growth and egg production as well as the magnitude and stoichiometry of the biological carbon pump^32^.

Silicates sinking below the depth of winter convection in the subpolar North Atlantic will either be deposited on the seafloor or drain equator-ward in the North Atlantic Deep Water – the lower limb of the Thermohaline Circulation system^33^. It therefore appears unlikely that changes in silicate inventories in the subpolar North Atlantic will significantly impact diatom productivity in other areas of the ocean.

It remains challenging to hindcast or forecast trends in silicate inventories beyond the 25 year record presented here. The observed silicate decline is primarily associated with the post-1995 weakening of the SPG^11^, and is therefore part of multi-decadal variability that observational records are too short to portray. The model simulations indicate that the silicate concentrations during the early 1960s, when winter convection and the SPG circulation were also weak^11^ would have been almost as low as they are today (Fig. 2b). The intensification in deep convection during the winters 2012–2016^34^ might have elevated the concentrations somewhat again. Nevertheless, over the next century, climate models predict global declines in nutrients and thus in primary production, due to shallower winter mixing, with a particularly strong imprint on the subpolar North Atlantic^35^. Our data shows that such climate scenarios may act in synergy with multi-decadal oscillations to regulate upper ocean nutrient inventories with important impacts on ecosystem productivity.

## Methods

### Primary datasets

For the *Nordic Seas* observation sites (S, M and G, Fig. 1), we have followed the methods in ref.^14^. The average silicate values for the upper water column every March were calculated using all observations either from 0–200 m or in the upper mixed layer, and only stations which sampled the Atlantic Water were included. In the central *Labrador Sea* (L in Fig. 1), the silicate records from all stations along the ARW7 line, except those on the shelves, were used. The section is occupied during May, and to avoid influence from the near-surface biology, only data from 150–500 m were included to produce Fig. 2a. The *Irminger Sea* (Ir in Fig. 1) silicate concentrations are the mean of 0–50 m, February-March values from a fixed station on the Faxaflói section, in the core of the Irminger Current.

### Supporting datasets

For the *Labrador Shelf* (LC in Fig. 1), the silicate concentrations have been averaged over the 50–150 m depth layer (Fig. 5). The *Iceland Basin* (Ic in Fig. 1) time-series was calculated by averaging all silicate samples between 150 m and 600 m from four stations along the 20°W portion of the Extended Ellett Line (EEL). Data from the *Faroe Shelf* (F in Fig. 1), represent winter (DJF) averages of all samples from a coastal station. The *Porcupine Abyssal Plain* (PAP, P in Fig. 1) dataset was sub-sampled from a ship of opportunity over a region close to the PAP site. For the southern *Bay of Biscay* (B in Fig. 1), silicate concentrations at the northern stations of the Santander standard section at 150 m depth are shown in Fig. 3. The Santander standard section has been sampled monthly since 1991.

### Ocean model

The model system applied in this study consists of the ocean general circulation model MPIOM (Max-Planck-Institute Ocean Model) and the marine biogeochemistry model HAMOCC (Hamburg Ocean Carbon Cycle Model). MPIOM is the ocean-sea ice component of the global earth system model MPI-ESM^36^ of the Max-Planck-Institute for Meteorology in Hamburg. For the presented simulation, the horizontal resolution in the northern North Atlantic ranges from around 13 km in the east and west to about 25 km over the Mid-Atlantic Ridge. In the vertical, the water column is resolved by 30 levels with 8 levels in the upper 100 m. Biogeochemical processes in the ocean are simulated by HAMOCC^37^, which is online coupled with MPIOM. Marine biology dynamics are represented by nutrients, phytoplankton, zooplankton, detritus, and dissolved organic matter^38^. The model simulation considered in this study covers the period 1958–2012. ERA40 reanalysis data^16^ are used as meteorological forcing at the sea surface for the period 1958–2000, continued by ERA-Interim data for the remaining period 2001–2012.

### The gyre index

The dynamics of the subpolar gyre has often been proxied by a so-called gyre index. This index is the principal component (no physical unit) obtained from an Empirical Orthogonal Function (EOF)^39^ analysis of the sea surface height field over the entire North Atlantic Ocean, obtained from satellite altimetry^13,21,40^ or from general circulation models^11,13^. We here utilize the gyre index from ref.^40^, which is based on a pre-2014 version of the Ssalto/DUACS altimetry products provided by the Copernicus Marine and Environment Monitoring Service (CMEMS) (http://www.marine.copernicus.eu). An updated gyre index based on the altimetry products released in April 2014 – the *DUACS 2014* – is available^41^. Changes in the satellite data processing in 2014 have, however, resulted in a slight change in the updated gyre index^21^.

### Statistics

The correlation coefficients are calculated by a standard Matlab routine which does not account for autocorrelation. The *P*-values are computed by transforming the correlation to create a *t*-statistic having *n*-2 degrees of freedom. (See Supplementary Information 1 for further details)^15,42–61^.

## Electronic supplementary material

Supplementary File
