# Supplementary material for: The subpolar gyre regulates silicate concentrations in the North Atlantic
Source: Sci Rep. 2017 Nov 6;7:14576. doi: 10.1038/s41598-017-14837-4 (PMC5674075; doi:10.1038/s41598-017-14837-4)
Supplement: Supplementary file 1 — Supplementary File [file 41598_2017_14837_MOESM1_ESM.pdf]

Supplementary Information for

The subpolar gyre regulates silicate concentrations in the North Atlantic

H. Hátún, J. Ólafsson, K. Azetsu-Scott, R. Somavilla, F. Rey, C. Johnson,
M. Mathis, U. Mikolajewicz, P. Coupel, J-É. Tremblay, S. Hartman, S.V. Pacariz and I. Salter.

correspondence to: hjalmarh@hav.fo

This PDF file includes:

1. Materials and Methods
2. Supplementary Text
3. Supplementary Figs. S1 to S7

1. Materials and Methods

Silicate observations (see also Methods in the main manuscript)

The silicate observations reported here are from several independent hydrographic sections crossing the Atlantic Inflow branches as well as the potential Arctic and subtropical water mass sources (Fig. 1). The individual *primary datasets* have been sampled in a consistent way and should be regarded as reliable. These are from the Labrador Sea (L in Fig.1), the Irminger Sea (Ir) and the Nordic Seas (S,M,G), all shown with colored squares in Fig. 1, and plotted in Fig. 2a. *Supporting datasets* are also utilized to support the primary datasets and to investigate the causes for the marked silicate decline. These are from the Iceland Basin (Ic), the Faroe Shelf (F), the Labrador Shelf (LC), the Porcupine Abyssal Plain (P) and the Bay of Biscay (B), shown with the black circles in Fig. 1

Data were obtained from: the Bedford Institute for Oceanography (BIO), Canada, the Marine Research Institute (MRI), Iceland, the Scottish Association for Marine Science (SAMS), UK, the Faroe Marine Research Institute (FAMRI), Faroe Islands, the Institute of Marine Research (IMR), Norway, the National Oceanography Centre (NOC), UK, and from the Spanish Institute of Oceanography (IEO). Below we provide further details to the Methods section in the main text.

Table S1 Overview over the silicate datasets

Location	Supplier	Depth (m)	Month	Comment
Labrador Sea (L)	BIO	150-500	May	Primary
Irminger Sea (Ir)	MRI	0-50	Feb-March	Primary
Nordic Seas (S,M,G)	IMR	0-200	March	Primary
Iceland Basin (Ic)	SAMS	150-600	Summer (varies)	Supporting
Faroe Shelf (F)	FAMRI	18	Dec-Feb	Supporting
Labrador Shelf (LC)	BIO	Hydr. criterion	May	Supporting
Porcupine Abyssal Plain (P)	NOC	Surface	March	Supporting
Bay of Biscay (B)	IEO	150 and 400	Every month	Supporting

In the central *Labrador Sea* (L, Fig. 1 and Table S1), all deep stations along the WOCE line ARW7, referenced in the Fisheries and Oceans Canada (DFO) BioChem oceanographic database (55.75-59.50°N, 53.50-49.50°W) were averaged from 150-500 m. The silicate concentrations were determined at the BIO using a Technicon Autoanalyzer II characterized by a 0.1 µM detection limit.

The *Irminger Sea* (Ir in Fig. 1 and Table S1) samples (64.33°N, 27.95°W) were analyzed for dissolved silicate with standard so-called colorimetric methods, and the long term accuracy has been estimated to ± 0.2 µM. Details of the analytical methods and quality assurance can be found in (ref. 42).

For the *Nordic Seas* observation sites (S, M and G, Fig. 1 and Table S1), samples were taken in the upper mixed layer. This is defined as the shallowest of either the upper 200 m, or the depths where the water density difference is less than 0.05 relative to the surface value[14]. Samples were only taken in Atlantic Water, identified by salinities greater than 35 and temperatures above 2°C.

The *Iceland Basin* (Ic in Fig. 1 and Table S1) time-series was calculated by averaging all silicate samples from four stations along the 20°W portion of the Extended Ellett Line (EEL) - latitude range 61.50 – 62.33°N (Fig. 4). Details of the analytical methods can be found in

(ref. 18). The EEL cruises are usually during the summer, but not at the same time each year. A station-wise rate of change analysis reveals an increasingly negative trend towards Iceland (Fig. S1)

For the *Faroe Shelf* (F in Fig. 1 and Table S1), the coastal station (Skopun, 61.91°N, 6.89°W) pumps water from 18 m depth and silicate samples have been collected twice a week since 1995. The nutrient samples were preserved with 12 drops of chloroform per 100 ml of seawater in polyethylene bottles and measured 3-6 months later according to (ref. 43).

Data for the *Canadian Arctic Archipelago passages* (Lancaster Sound, Smith Sound and Hudson strait, Fig. S6) come from the annual Arctic Net cruises (1998-2013) conducted on-board the icebreaker Amundsen between July and October each year. The sampling and analysis of the silicate concentrations were performed at University Laval according to the method described in (ref. 8). Data prior to 1998 come from the ICES Data Centre (http://ices.dk/).

For the *Labrador Shelf* (LC, Fig. 1 and Table S1), the silicate concentrations have been averaged over the depth layer 50 -150 m, to avoid the impact of near-surface biological activity (Fig. 5).

The *Porcupine Abyssal Plain* (PAP, P in Fig. 1 and Table S1) sustained observatory site is described in (ref. 44). For comparisons with sensors at the site, surface nutrient data was collected from a ship of opportunity on the UK-Caribbean route[45]. This data set was sub sampled for the region 45-52°N, 26.08-8.92°W to be close to the PAP site. On the UK-Caribbean ship of opportunity route samples were collected and frozen on-board each month for analysis ashore using a Skalar auto-analyser[46]. Working standards were compared with standards prepared by Ocean Scientific International (OSI) to ensure the results were consistent from month to month. The precision obtained was assessed through duplicate measurements as 0.1%.

For the southern *Bay of Biscay* (B in Fig. 1 and Table S1), silicate concentrations at the northern station of the Santander standard section (150 m, 43.80ºN, 3.78ºW, 2580 m bottom depth) is representative of the winter silicate concentration in the mixed layer (Fig. 3). The samples for silicate were frozen and subsequently concentrations determined with a Technicon autoanalyzer. For comparison these are plotted together with the near-surface winter (March) silicate concentrations around the PAP mooring (see above) (Fig. 3).

Ocean model description (see also Methods in the main manuscript)

The primitive equations of oceanic motion in the MPIOM are discretized on an Arakawa C-grid with z-coordinates and free surface, including hydrostatic and Boussinesq approximations. Tracer and momentum advection follows a second order total variation diminishing scheme after (ref. 47). Vertical mixing is performed after (ref. 48) with an additional parameterization for wind stirring[39]. In the model version described here, the full ephemeridic luni-solar tidal potential is included according to (ref. 49). A detailed model description and evaluation is given in (refs. 50,51). For the specific application to the northern North Atlantic region, a stretched grid configuration has been used with non-diametrical poles located in southwest Germany and Wisconsin (USA).

Biogeochemical cycles and trophic levels in HAMOCC are connected by nutrient uptake and remineralization of organic matter, including co-limitation by major macro and micro nutrients, such as phosphate, nitrate, and dissolved iron[52, 53]. A sediment module accounts for deposition processes of solid constituents as well as for the biogeochemistry of pore water tracers in the upper bioturbated sediments[53]. Resuspension in the model configuration described here is implemented after (ref. 54). Tracer transport is simulated by MPIOM.

The simulation has been initialized by a spin-up run of 120 years with repeated forcing of the first 20 years of ERA40 data (1958-1977). River runoff is prescribed as monthly climatological means, calculated by the Hydrological Discharge model[55].

Additional funding information

The acquisition and compilation of silicate data from the western Arctic was supported by the Network Center of Excellence ArcticNet and Fisheries and Oceans, Canada, Atlantic Zone Offshore Monitoring Program (AZOMP). The captains and the crew of *CCGS Hudson,* chief scientists and scientific parties of AZOMP are acknowledged. The analysis of silicate data from the Labrador Sea was supported by a grant from the National Sciences and Engineering Research Council of Canada to the CCAR network VITALS (Ventilation and Transports across the Labrador Sea).

The EEL is funded through UK NERC's National Capability program.

The nutrient analysis of the Santander standard section has been performed in relationship with the project Radiales funded by the Spanish Institute of Oceanography.

The PAP sustained observatory work was supported through the Natural Environment Research Council (NERC), UK, project Oceans 2025 and National Capability. The PAP-SO and ship of opportunity in that region also contribute to the EU funded FixO3 project EU 312463 and the NERC Greenhouse Gas TAP NE/k00249x/1.

2. Supplementary Text

Validation of HAMOCC

The simulated silicate concentrations during April are comparable to World Ocean Atlas climatology (Fig. S2). A clear separation line between subpolar and subtropical water masses aligns with the North Atlantic Current and maximum concentrations are found in the Labrador Sea convections site, while elevated concentrations embed the northern Irminger Sea and Iceland Basin. The simulated concentrations in the Baffin Bay are, however, much lower than the representation in the World Ocean Atlas. Also note that the World Ocean Atlas shows highest silicate concentrations in the Irminger Sea region during March (Fig. S2a), followed by maximum values in the Labrador Sea during April (Fig. 2Sb).

In addition, primary production is in good agreement with satellite data presented in (ref. 56), with about 580 mg C m^{-2} d^{-1} in the Central Irminger Sea, 650 mg C m^{-2} d^{-1} in the Iceland Basin and 750 mg C m^{-2} d^{-1} at Reykjanes Ridge during July to September 1998-2010. Moreover, observed annual cycles in the Irminger Sea of near-surface nitrate concentrations as well as phytoplankton and zooplankton abundances presented in (ref. 57) for the years 2001 and 2002 are well reproduced by the model, with winter nitrate concentrations ranging between 10 and 14 µM.

A linear regression analysis of the simulated near-surface (0 – 150 m) March silicate at each HAMOCC model grid for the period 1990-2011 reveals a significant negative trend in the following regions: *i*) At the entrance to the Rockall Trough (west of the British Isles) where several water masses with strongly contrasting properties meet, *ii*) along the periphery of the SPG through the Iceland Basin, the Reykjanes Ridge, the Irminger Sea and the Labrador Sea, *iii*) the south Iceland Shelf, *iv*) along the sub-arctic front in the Nordic Seas and *v*) in a region in the central SPG, south of Greenland (Fig. S3). This is likely due to the westward retracting SPG during this period[11], and the associated replacement of silicate rich subarctic waters with

silicate poorer Atlantic waters along the retracting subarctic front. A similar westward retraction of the subarctic front in the Nordic Seas would also induce a silicate decline near the frontal zone there. Periods with weak atmospheric forcing result in both a declining SPG[11, 12] and a westward shift of the subarctic front in the Nordic Seas[58].

The HAMOCC model shows very strong silicate variability in the central SPG since the late 1950s, which closely follows the gyre index from (ref. 11), based on output from the Miami Isopycnal Coordinate Ocean Model (MICOM) (Fig. S4). The negative trend in the central SPG (54-56°N, 48-45°W, blue blob south of Greenland in Fig. S3) is primarily caused by an extreme drop of nearly 6 µM during the years 1995-1999, when the gyre suddenly declined[11].

HAMOCC, however, does not show a significant decline along the WOCE line ARW7 (Fig. S3) where the observations show a clear silicate decrease (Fig. 2 and Table 1). It is well known that such model systems can produce realistic temporal variability associated with deep convection, but they seldom correctly identify the location of real convection regions. The general circulation model MPIOM, which is the physical component of HAMOCC, has such limitations, and this could explain the likely spurious spatial pattern within the central SPG.

Arctic silicate flux estimate

Since 1997, the Arctic system has been under the influence of an anticyclonic circulation regime, described as a positive phase of the Arctic Ocean Oscillation[26]. Under these conditions, wind forcing leads to freshwater (FW) accumulation in the Arctic Ocean through Ekman convergence and thus a reduction of the FW flux to the North Atlantic subpolar regions (Fig. S7b). Most of the FW accumulation occurs in the Beaufort Gyre, where not only Pacific Water (PW) rich in silica accumulates, but also FW from river runoff[59] with even higher silicate concentrations[60] converges. Estimates of the FW build up in the Beaufort Gyre since the mid-1990s range between 8,000 km^3 and 12,000 km^3 (refs. 28, 61). Knowing the silicate concentrations representative of this FW enables us to estimate the associated reduction in Arctic silicate flux to the subpolar North Atlantic Ocean.

Silicate concentration representative of the FW accumulated in the Beaufort Gyre.

Estimations of FW accumulation in the Arctic are made with respect to the 34.8 isohaline, typically located within the upper 350 m depth in the Canada Basin. Above it (50-250 m depths), a silicate maximum (~35 µM) is observed, associated with the PW (e.g. Fig. 7 in ref. 9). The PW halocline is found between the PW core and the 34.8 isohaline, with lower but still high silicate concentrations.

On its way from the Canada Basin through the Canadian Arctic Archipelago to Davis Strait, homogenization of the Pacific-origin waters occurs as noted by (ref. 8). Similar total levels of salt and silicate are found in the upper 150 m for Silicate-Rich Arctic Water (SRAW) in Baffin Bay and the Canada Basin. Thus, the silicate concentration in the homogenized SRAW (~22 µM)[8] is assumed to be representative of the Pacific-origin waters in the Canada Basin. In order to estimate the silicate accumulation in the Beaufort Gyre ($\sum Si_{BG}$), a reference silicate concentration, here represented by the mean concentration of the Arctic, must be subtracted from the SRAW concentration. Using the Pangaea data set (https://www.pangaea.de/), we get Si_{ref} ~11 µM. This gives:

$$\sum Si_{BG}(I) = FW_{BG}\ [8000\text{-}12000\ km^3] \cdot (22\ \mu M - 11\ \mu M)$$
$$= -\underline{[8.8\text{-}13.2] \cdot 10^{16}\ \mu mol}$$

Alternatively, we could estimate $\sum Si_{BG}$ from the silicate maximum of the PW, previously estimated to contribute 50% to the total FW content in the Beaufort Gyre[62].

$$\sum Si_{BG}(II) = 0.5 \cdot FW_{BG}\ [8000\text{-}12000\ km^3] \cdot (35\ \mu M - 11\ \mu M)$$
$$= -\underline{[9.6\text{-}14.4] \cdot 10^{16}\ \mu mol}$$

Contribution to the subpolar North Atlantic.

According to (ref. 9), between 85 and 100% of the silicate in the Arctic leaves the basin through the Canadian Arctic Archipelago, mainly in the density range $\sigma_\theta = 27.0 – 27.5$. Based on the climatological distribution of the 27.0 and 27.5 isopycnals in the North Atlantic (including the Labrador and Irminger seas), we estimate the volume into which the SRAW potentially would redistribute: $V_{NAtl} = 4.11 \cdot 10^{14}\ m^3$.

The silicate decrease in the subpolar North Atlantic, caused by the retention of silicate in the Arctic Ocean (assuming that 90% reaches the North Atlantic) is therefore:

$$\Delta Si_{NAtl}\ (I) = 0.90 \cdot \sum Si_{BG}(I) / V_{NAtl} = -0.90 \cdot [8.8\text{-}13.2] \cdot 10^{16}\ \mu mol / 4.11 \cdot 10^{14}\ m^3$$
$$= -\underline{(0.19\text{-}0.29)\ \mu M}$$

$$\Delta Si_{NAtl}\ (II) = 0.90 \cdot \sum Si_{BG}(II) / V_{NAtl} = -0.90 \cdot [9.6\text{-}14.4] \cdot 10^{16}\ \mu mol / 4.11 \cdot 10^{14}\ m^3$$
$$= -\underline{(0.21\text{-}0.33)\ \mu M}$$

Thus, the accumulation of SRAW in the Beaufort Gyre and Canada Basin could explain up to 10-20% of the total silicate decline observed in the subpolar North Atlantic (1.5-2 μM). A more realistic estimate must, however, include changes in FW transport into the Arctic through the Bering Strait.

FW transports through Bering Strait.

The annual FW transport through the Bering Strait has increased from 2000–2500 km^3 in 2001 to 3000-3500 km^3 in 2011[29]. Assuming a linear increase as observed in ref. 29 (100 km^3 per year over ten years), this would result in a FW increase of 5000 km^3 from 2001 to 2011, which is about half of the observed FW accumulated in the Beaufort Gyre.

On the other hand, a 26% decrease in the FW transport through Davis Strait from 4500 ± 730 km^3 for the period 1987-1990 to 3300 ± 220 km^3 for the period 2004-2010 would explain up to 10,000 km^3 of FW accumulation in the Arctic[52]. Together, they would result in a FW accumulation of 15,000 km^3, ~1/3 contribution from Bering Strait FW transports and ~2/3 from Davis Strait FW transports.

Thus accounting for the increased Bering Strait FW contribution to the observed Beaufort Gyre FW accumulation since the mid-1990s, we estimate the Arctic contribution to the North Atlantic silicate decline (1.5-2 μM, Fig. 2a) to be less than 15%.

3. Supplementary Figures

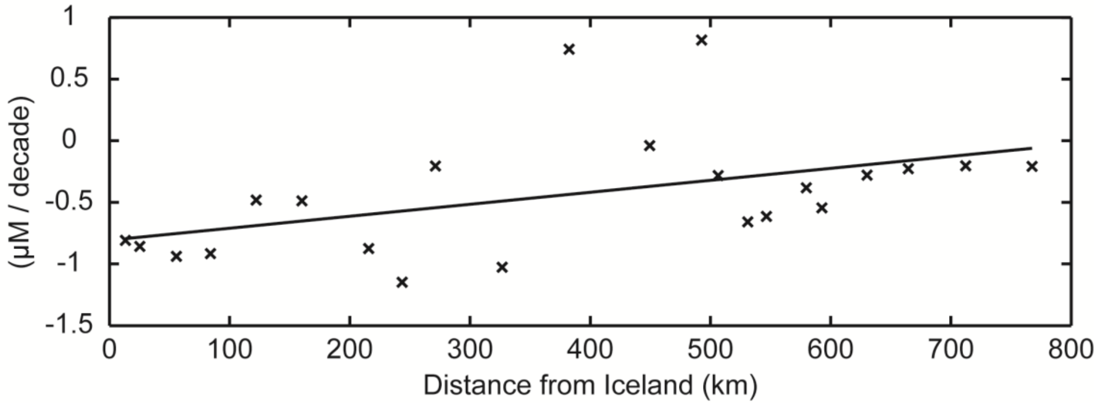

Fig. S1 Rate of change in silicate concentrations between 150-600 m from 1997 to 2015 for each EEL station between Iceland (0 km) and Rockall (800 km) (see Fig. 4). Only stations where data from more than five cruises are available have been included. The rate of change has been calculated by a least square analysis.

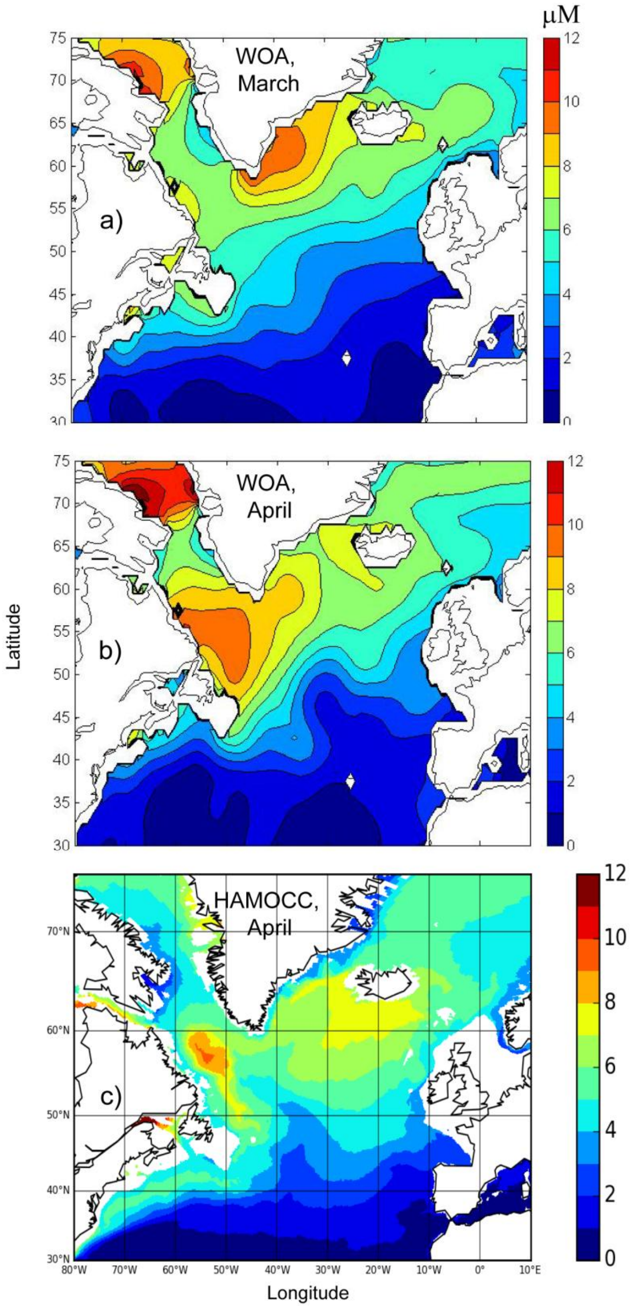

Fig. S2 Climatological upper-ocean (average over 0-200 m) silicate concentrations. Panels **a**) and **b**) show the representation in the World Ocean Atlas (WOA[20]) for March and April, respectively and **c**) shows the simulated values from HAMOCC for April (1960-2012). The figure was produced using the softwares Matlab R2013b, (https://www.mathworks.com) (panels **a** and **b**) and the Python programming language (Python Software Foundation. Python Language Reference, version 2.7, available at http://www.python.org).

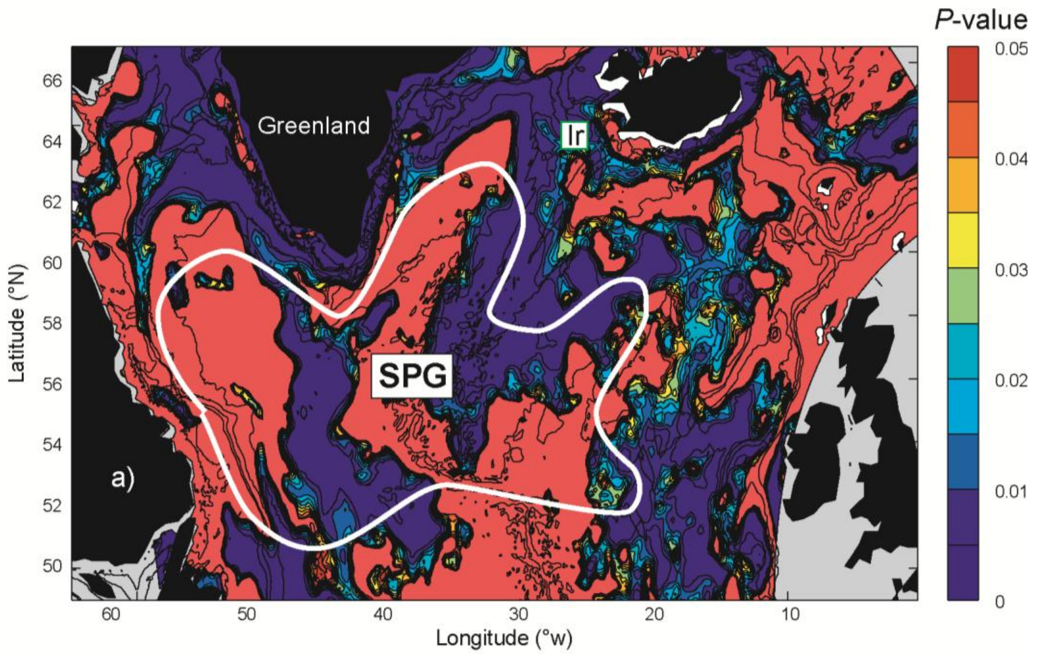

Fig. S3 Linear trend in the simulated (HAMOCC) near-surface (0 – 150 m) silicate during March. The colors show *P*-values from a linear regression analysis of the silicate at each model grid for the period 1990-2011. The blue colors represent the regions where a declining trend is simulated, and the areas colored red show no clear trend. The rough outline of the subpolar gyre (SPG) is depicted with the white curve. The figure was produced using the software Matlab R2013b, (https://www.mathworks.com).

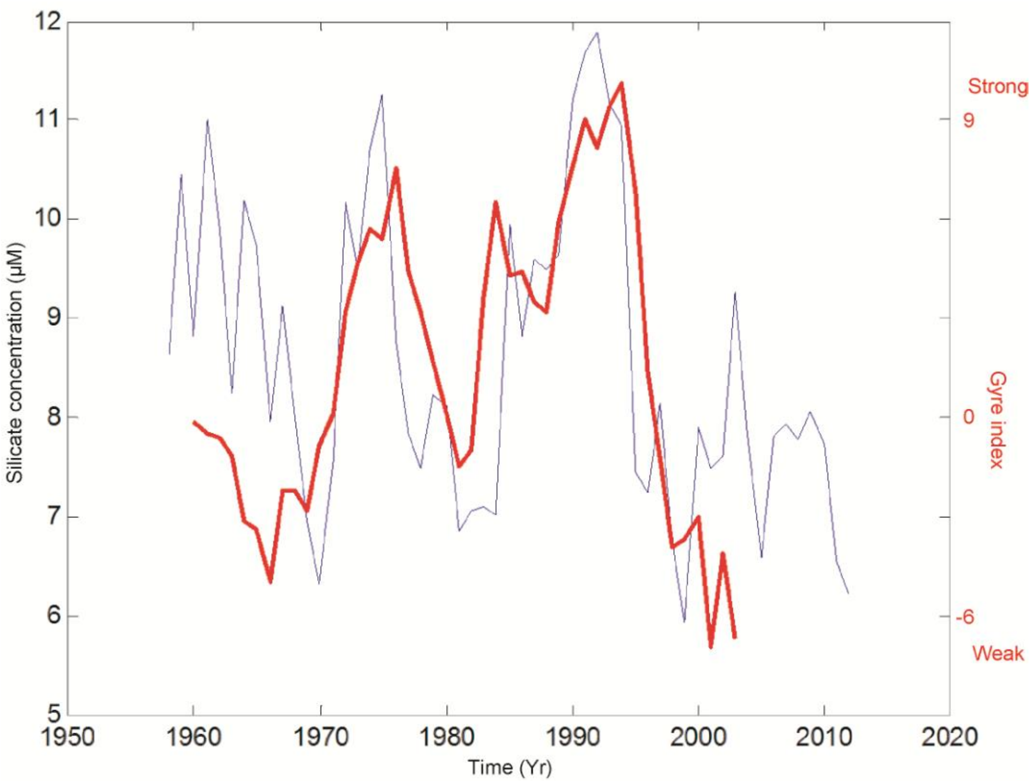

Fig. S4 Simulated near-surface (0-200 m) March silicate concentration (HAMOCC) in the central SPG (54-56°N, 48-45°W) and the gyre index presented by (ref. 11) (red), which was based on the Miami Isopycnal Coordinate Ocean Model (MICOM).

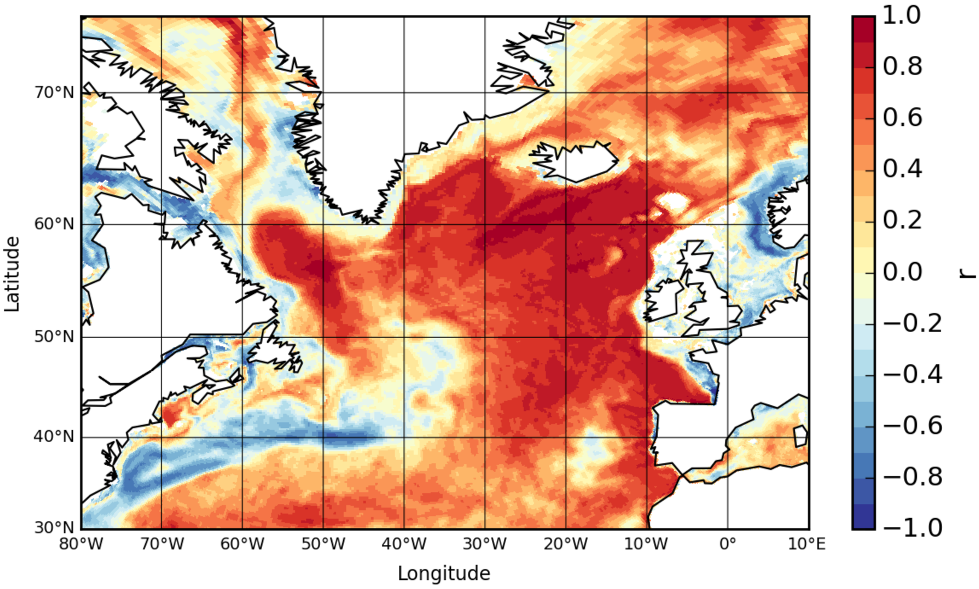

Fig. S5 Correlations between the simulated winter (DJF) near-surface silicate concentrations, and the mixed layer depth (DJF) at every model (HAMOCC) grid-point for the years from 1958 to 2012. The figure was produced using the Python programming language (Python Software Foundation. Python Language Reference, version 2.7, available at http://www.python.org).

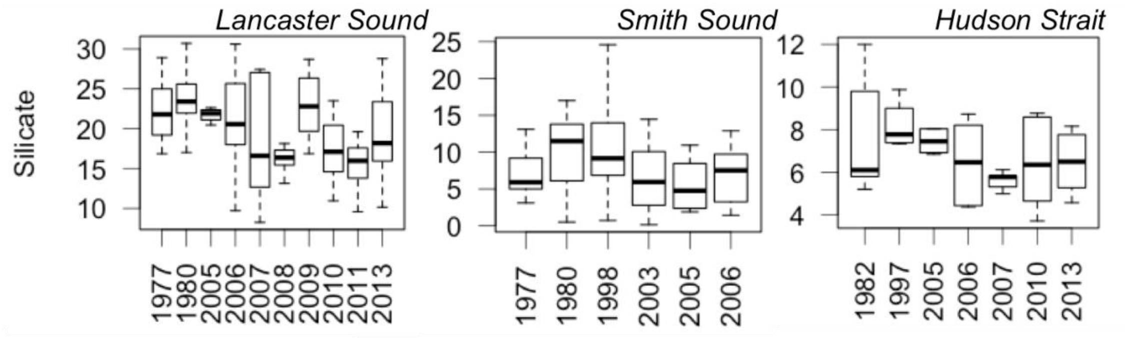

Fig. S6. Temporal changes of the silicate concentration (µM) in the Arctic Outflow at three passages through the Canadian Arctic Archipelago: Lancaster Sound (50-150 m), Smith Sound (0-100 m) and Hudson Strait (50-150 m).

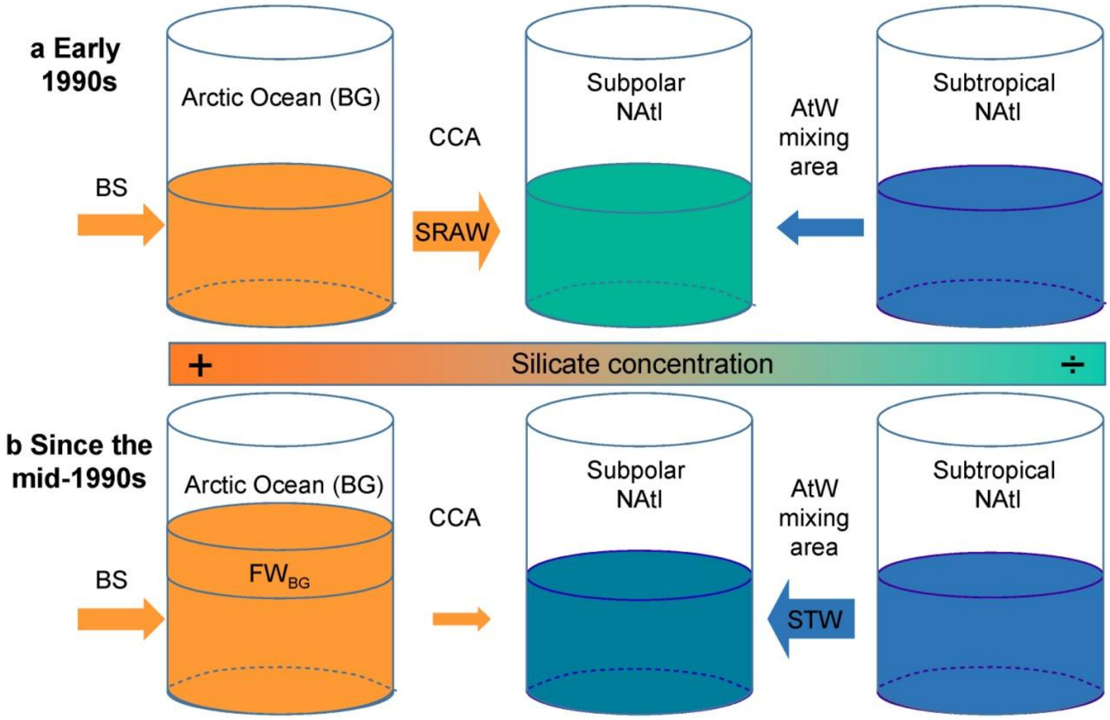

Fig. S7. Schematic diagram of Silicate-Rich Arctic Water (SRAW) and subtropical Water (STW) contributions to the silicate content in the subpolar North Atlantic. a) The situation at the beginning of the 1990s, after the large loss of Arctic Freshwater content and before the beginning of large fresh water accumulation in the Beaufort Gyre (BG) since the mid-1990s. **b**) After the mid-1990s, with SRAW of Pacific origin accumulation in the BG (FW_{BG}) through Ekman convergence and reduction of SRAW flux to the North Atlantic Subpolar regions. BS, Bering Strait; CAA, Canadian Arctic Archipelago.
